# Epigenetic Regulation of DNA Methylation and RNA Interference in Gastric Cancer: A 2024 Update

**DOI:** 10.3390/biomedicines12092001

**Published:** 2024-09-03

**Authors:** Iulia Lupan, Vasile Bintintan, Diana Deleanu, Gabriel Samasca

**Affiliations:** 1 Department of Molecular Biology, Babes-Bolyai University, 400084 Cluj-Napoca, Romania; iulia.lupan@ubbcluj.ro; 2 Department of Surgery 1, Iuliu Hatieganu University of Medicine and Pharmacy, 400006 Cluj-Napoca, Romania; vasile.bintintan@umfcluj.ro; 3 Department of Immunology, Iuliu Hatieganu University of Medicine and Pharmacy, 400006 Cluj-Napoca, Romania; deleanu@umfcluj.ro

**Keywords:** gastric cancer, epigenetics, long noncoding RNAs, m6A modification, therapeutic targets

## Abstract

Gastric cancer (GC) remains a significant public health concern because of its lethality, underscoring the need for deeper insights into its molecular mechanisms. Recent studies have increasingly highlighted the role of epigenetic modifications as critical players in cancer progression. Despite their importance, research specifically addressing epigenetic factors in GC is relatively scarce. This paper seeks to bridge that gap by examining recent literature that elucidates the epigenetic landscape associated with GC. The investigation of long noncoding RNAs (lncRNAs) has revealed their substantial involvement in gene dysregulation and epigenetic alterations within GC tumors. Notably, lncRNAs such as LINC00853 and LINC01266 have been identified as significant contributors to the epigenetic modulation of gene expression. Furthermore, the overexpression of *KAT5* and *GPX4* has been shown to mitigate the antiproliferative effects resulting from the depletion of circRHOT1, suggesting a complex interplay between these molecules in GC pathophysiology. Another pivotal aspect of epigenetic regulation in GC involves modifications in N6-methyladenosine (m6A), which play crucial roles in mRNA maturation processes such as splicing, export, degradation, and translation. m6A modifications are known for their influence on various cancer-related pathways, thus presenting a potential avenue for targeted interventions. Our findings indicate that the most pronounced instances of epigenetic dysregulation in GC can be traced back to the effects of long lncRNAs and alterations in m6A modification patterns. This underscores the urgent need for comprehensive investigations into these epigenetic factors, as a deeper understanding could lead to enhanced diagnostic markers and innovative therapeutic strategies. The integration of genetic and epigenetic considerations is essential for advancing the field of GC research. This synthesis of recent findings concerning epigenetic regulation offers valuable insights that could inform future studies and therapeutic developments. There is a critical need for ongoing research to elucidate the complexities of epigenetic modifications in GC, ultimately improving patient outcomes through tailored interventions.

## 1. Introduction

Epigenetic mechanisms are essential in the intricate regulation of gene expression, influencing a myriad of biological processes through modifications that occur without altering the underlying DNA sequence. Among the key epigenetic processes are chromatin remodeling, covalent modifications of histone proteins, and the dynamic regulation of DNA methylation and demethylation. These mechanisms collectively orchestrate the accessibility of genetic material and subsequently modulate transcriptional activity. Importantly, the dysregulation of these epigenetic processes has gained attention as a significant clinical marker, particularly concerning metastasis, disease recurrence, and oncogenesis. Understanding the role of epigenetics in these contexts may provide valuable insights into the development of targeted therapeutic strategies and enhanced diagnostic tools in the realm of clinical oncology. The interplay between epigenetic modifications and gene expression underscores the complexity of cancer biology and highlights the need for further research to elucidate the potential of epigenetic interventions in improving patient outcomes [1].

The increasing prevalence and mortality rates associated with gastric cancer (GC) highlight its emergent importance as a pressing global health concern. The pathophysiology of GC is multifaceted and involves a complex interplay between environmental influences and genetic susceptibilities. Consequently, elucidating novel genes and pathways implicated in the initiation and advancement of GC is crucial for enhancing diagnostic accuracy and therapeutic effectiveness. By advancing our understanding of the molecular mechanisms underlying this malignancy, we can pave the way for the development of targeted interventions, ultimately improving patient outcomes and reducing the burden of GC on healthcare systems worldwide [2].

In the present study, our objective was to compile and critically analyze the most recent advancements in the epigenetic regulation of GC. To accomplish this, we conducted a systematic search of the PubMed database utilizing the keywords “Epigenetic Regulation” and “Gastric Cancer Progression”. Our search criteria were strictly limited to studies published between 2023 and 2024 to ensure that our analysis reflects the latest research developments in the field. Importantly, our research design deliberately excluded case reports. This methodological choice was made to maintain the quality and relevance of our findings, thereby enhancing the reliability of our conclusions regarding the epigenetic mechanisms influencing GC progression.

**Figure 1:**
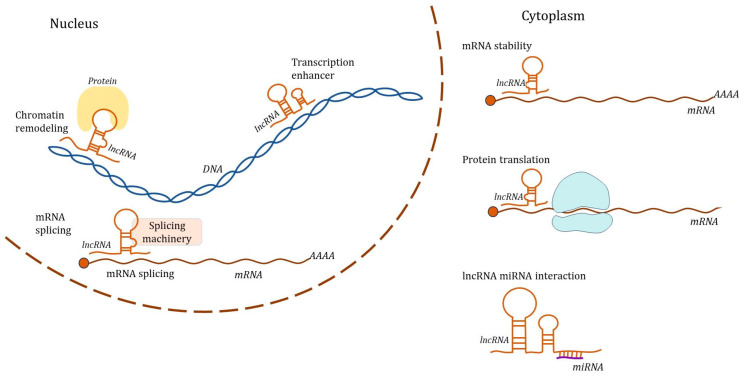
General functions of lncRNAs in the regulation of gene expression.

The analysis conducted elucidated several pivotal areas of inquiry pertaining to the role of epigenetic regulation in the progression of GC. Key findings highlighted the importance of long noncoding RNAs (lncRNAs) and circular RNAs (CircRNAs) as critical regulators in this context. Furthermore, alterations in N6-methyladenosine (m6A) methylation were identified as substantial factors influencing GC pathogenesis. Experimental investigations employing various GC cell lines, alongside both in vivo and in vitro methodologies, have provided robust insights into the underlying mechanisms at play. Notably, the integration of machine learning techniques facilitated the identification of patterns and correlations within large datasets, whereas a network-based integrative approach enabled a comprehensive understanding of the multifaceted interactions involved in GC. Additionally, an analysis of the immunological landscape surrounding GCs has shed light on the complex interplay between cancer and the immune system. This research also highlights potential biomarkers that could serve as diagnostic or prognostic indicators for gastrointestinal tumors and various other cancers, underscoring the importance of epigenetic factors in cancer biology. These findings not only advance our understanding of GC but also pave the way for future therapeutic strategies targeting epigenetic modifications (Figure 1).

## 2. IncRNAs and CircRNAs

Among lncRNAs, long intergenic nonprotein coding RNA 853 (LINC00853) has been demonstrated to be significantly overexpressed in GC tissues, facilitating the movement, invasion, and proliferation of GC cells in a study by Xu et al. [3]. Another lncRNA of interest, long intergenic nonprotein coding RNA 1266 (LINC01266), is a novel carcinogenic lncRNA transcribed from the 1p35.2 genomic region, a well-established risk locus for GC. Notably, in a study by Hua et al., the expression levels of *LINC01266* in GC samples were markedly elevated compared with those in adjacent normal tissues [4]. The pathogenesis of GC is intricately linked to gene dysregulation and epigenetic modifications, which contribute significantly to tumor development. Within this context, new oncogenes such as ASH1-like histone lysine methyltransferase (*ASH1L*) and its corresponding antisense lncRNA, *ASH1L-AS1*, emerge from the prominent GC risk signal located at 1q22. In a study by Xie et al., elevated expression levels of *ASH1L* or *ASH1L-AS1* in GC specimens were associated with poorer patient prognoses [5].

In a comprehensive analysis by Askari et al., differentially expressed mRNAs (DEmRNAs) and lncRNAs (DElncRNAs) were identified from three GC tissue microarray datasets. This analysis revealed a total of 856 DEmRNAs—comprising 451 upregulated and 405 downregulated transcripts—and 9 DElncRNAs, highlighting the distinct expression profiles between tumor samples and nearby normal tissues. These findings contribute to the growing body of evidence supporting the critical involvement of lncRNAs in the molecular mechanisms underlying GC and present opportunities for future therapeutic strategies. Six differentially expressed DElncRNAs were identified that are predicted to interact with a total of one hundred and seventeen differentially expressed messenger RNAs (mRNAs), including key transcripts such as H19 Imprinted Maternally Expressed Transcript (H19), WT1 transcription factor (WT1)-AS, EMX2 opposite strand/antisense RNA (EMX2OS), HOX transcript antisense RNA (HOTAIR), ZEB1 antisense RNA 1 (ZEB1-AS1), and long intergenic nonprotein coding RNA 261 (LINC00261) [6].

The role of circRNAs has been extensively studied by Wang H. et al., particularly concerning their regulatory functions in cancer development and progression. Notably, circ Ras homolog family member T1 (RHOT1) is expressed at significantly higher levels in GC tumors than in adjacent nontumor tissues. The knockdown of circRHOT1 led to a significant reduction in cell growth (*p* < 0.05) and promoted ferroptosis in GC cells (*p* < 0.05). Mechanistically, circRHOT1 appears to facilitate the recruitment of lysine acetyltransferase 5 (KAT5), which subsequently promotes the acetylation of lysine 27 on the histone H3 protein subunit (H3K27Ac) associated with the glutathione peroxidase 4 (GPX4) gene, thereby increasing its transcriptional activity. Furthermore, the overexpression of *KAT5* and *GPX4* has been shown to counteract the antiproliferative effects induced by the depletion of circRHOT1 (*p* < 0.05) [7].

The ongoing exploration of biomarkers in oncology has led to the identification of various molecular entities that exhibit commonality across multiple cancer types. One such biomarker is zinc finger antisense 1 (ZFAS1), a recently identified lncRNA that has garnered attention for its oncogenic potential in human malignancies. *ZFAS1* is notably overexpressed in a range of cancers, including colorectal cancer (CRC), colon cancer, osteosarcoma, and GC, indicating its possible role as a universal marker for tumorigenesis. Research has shown that ZFAS1 is intricately involved in critical cellular processes such as apoptosis, cell proliferation, the cell cycle, and cellular migration. Additionally, it plays a role in translation, ribosomal RNA (rRNA) processing, and the assembly of spliceosomal small nuclear ribonucleoproteins (snRNPs). The regulatory functions of ZFAS1 include the modulation of various signaling pathways, where it interacts with transcription factors through binding to pivotal proteins and microRNAs (miRNAs). However, importantly, the literature presents conflicting findings regarding the precise impacts of ZFAS1 on these cellular processes, underscoring the complexity of its role in cancer biology. As the scientific community continues to investigate the multifaceted functions of ZFAS1, its potential as a prognostic biomarker and therapeutic target remains an area of significant interest, as it is promising for enhancing our understanding of cancer pathogenesis and treatment strategies [8]. Recent research has illuminated the potential translational roles of circRNAs, indicating a shift in our understanding of their function beyond mere regulatory elements. Notably, cap-independent translation of circRNAs is facilitated by internal ribosome entry sites (IRESs) and m6A modifications. To substantiate the hypothesis of circRNA translation, a green fluorescent protein (GFP)-containing circRNA minigene reporter system was employed, which successfully demonstrated the translational capability of these unique RNA structures. Moreover, various circRNA-encoded proteins and peptides—such as circFBXW7, circFNDC3B, circLgr4, circPPP1R12A, circMAPK1, circβ-catenin, circGprc5a, circ-SHPRH, circPINTexon2, and circAKT3—have been linked to human malignancies, including GC, hepatocellular carcinoma (HCC), bladder cancer, triple-negative breast cancer, colon cancer, and glioblastoma. Collectively, these findings underscore the significant role that circRNAs may play in the translation processes of the human genome, suggesting their potential implications in oncogenesis and cancer progression. This emerging understanding opens new avenues for further research into circRNAs as critical contributors to cellular function and pathology [9].

Regarding the therapeutic potential of targeting lncRNA molecules in GC treatment, lncRNAs, circRNAs, and miRNAs have emerged as key players in the regulatory networks that govern cancer cell behavior. They are implicated in various aspects of GC, including cell proliferation, apoptosis, invasion, and metastasis. For example, certain lncRNAs promote tumor growth by modulating signaling pathways, whereas specific miRNAs can act as tumor suppressors or oncogenes, depending on the context of their expression. Furthermore, the dysregulation of these noncoding RNAs has been associated with unfavorable clinical outcomes and resistance to conventional therapies. The therapeutic targeting of noncoding RNAs represents a novel approach to GC treatment. Strategies may include the use of antisense oligonucleotides, small interfering RNAs (siRNAs), or CRISPR(clustered regularly interspaced short palindromic repeats)-based technology to inhibit the function of specific lncRNAs or miRNAs that drive tumorigenesis. Additionally, restoring the expression of tumor-suppressive noncoding RNAs could reverse malignant phenotypes and enhance chemosensitivity. Such targeted approaches offer the potential to improve patient outcomes by addressing the underlying molecular drivers of GC. One of the significant advantages of noncoding RNAs is their presence in bodily fluids, such as serum, where they can serve as noninvasive biomarkers. The detection of circulating RNA molecules could provide valuable insights into the prognosis and therapeutic response of GC patients. The research indicates that specific profiles of serum lncRNAs, circRNAs, and miRNAs are correlated with disease stage and treatment outcomes, suggesting their utility in guiding clinical decision-making. The exploration of noncoding RNAs as therapeutic targets in GC represents a promising frontier in oncology. By harnessing the regulatory potential of lncRNAs, circRNAs, and miRNAs, researchers and clinicians can pave the way for innovative treatment modalities that increase the effectiveness of existing therapies and improve patient prognoses. Additionally, the ability to utilize circulating noncoding RNA molecules as noninvasive biomarkers holds significant promise for advancing the precision medicine paradigm in GC management. Continued research in this domain is essential for translating these findings into clinical practice, ultimately improving outcomes for patients afflicted with this challenging disease [10].

These findings underscore the significant regulatory roles that specific noncoding RNAs play in the molecular mechanisms underlying GC.

## 3. m6A Alteration

Recent advancements in molecular biology have highlighted the critical role of m6A modification in various biological processes, particularly in the context of cancer. Wang et al. suggested that alterations in m6A play pivotal roles in the splicing, export, degradation, and translation of mRNAs, influencing cancer pathophysiology. The expression patterns of m6A regulators are heterogeneous across different cancer types and are intricately regulated by mRNAs and noncoding RNAs, with lncRNAs being particularly significant. As the most prevalent and consequential chemical modification of RNAs, m6A is increasingly recognized for its epigenetic implications in cancer development, leading to a surge of interest in the field of epigenetics [11].

Among the various m6A demethylases identified, fat mass and obesity-associated protein (FTO) stand out as the first discovered enzymes in this category. Notably, clinical data derived from Kaplan–Meier plots indicate a strong correlation between elevated levels of FTO and poor patient prognosis in cancer patients. Experimental investigations conducted by Wu et al., both in vitro and in vivo, have substantiated the ability of FTO to promote the proliferation of GC cells. Mechanistically, FTO interacts with circFAM192A, preventing its degradation by specifically binding to it and removing its m6A modification. This interaction subsequently stabilizes the leucine transporter solute carrier family 7 member 5 (SLC7A5), which is influenced by circFAM192A. The increased presence of SLC7A5 on the cell membrane enhances leucine uptake, thereby activating the mammalian target of the rapamycin (mTOR) signaling pathway. These findings underscore the intricate relationships among m6A modification, FTO activity, and the regulatory networks that govern cancer cell metabolism and growth. For these experiments, the following methods were used: cell culture, quantitative real-time PCR (qRT–PCR), plasmid/siRNA transfection and lentiviral transduction, RNA stability assay, RNA fluorescence in situ hybridization (FISH), ethynyl-2′-deoxyuridine (EdU) incorporation assay, Western blotting (WB), Northern blotting (NB), dual-luciferase reporter assay, measurement of the Leu concentration, RNA–protein immunoprecipitation (RIP), RNA pull-down, animal studies, and bioinformatics analysis [12].

The initiation of GC is intricately associated with m6A RNA methylation, a crucial epigenetic process that plays a significant role in cellular regulation. Notably, exposure to methylnitronitrosoguanidine (MNNG) has been identified as a pivotal factor influencing this methylation. Recent investigations by Liu et al. into lncRNAs linked to MNNG exposure have yielded insightful results. Through methylated RNA immunoprecipitation sequencing (MeRIP-seq), a total of 3520 lncRNA transcripts exhibiting altered m6A methylation patterns were detected in GC cells, with 1432 lncRNA transcripts specifically expressed in malignant cells (MCs). The process of N6 methylation of lncRNAs is mediated primarily by the N6-adenosine-methyltransferase 70 kDa subunit (METTL3). Furthermore, small nucleolar RNA host gene 7 (SNHG7) has been established as a downstream target of METTL3, with MNNG exposure resulting in the progressive upregulation of SNHG7 throughout the gastric carcinogenesis process. For these experiments, the following methods were used: public GC data analysis, clinical specimens and data, cell lines and cell culture transfection, MNNG-induced malignant transformation of GES-1 cells, quantitative real-time PCR (qRT–PCR) and plasmid transfection, Western blotting analysis, RNA extraction and qRT–PCR, scratch wound healing assays, cell invasion assays, MeRIP-Seq, functional enrichment of target lncRNAs and statistical analyses [13].

In a study conducted by Zhang et al., the role of METTL3 within the context of head and neck squamous cell carcinoma (HNSCC) was elucidated, highlighting its contribution to cellular proliferation. The authors demonstrated that METTL3 enhances cell proliferation through its interaction with the methyltransferase METTL1, which is crucial for modulating the m7G modification of the cell cycle regulator CDK4. This interaction results in the stabilization of CDK4, as METTL1 physically binds to its transcript, thereby influencing the level of m7G modification. Notably, the results indicate that the overexpression of *CDK4* significantly mitigates the inhibitory effects associated with *METTL1* knockdown. These findings underscore the pivotal role of CDK4 in the proliferation dynamics of various malignancies, including endometrial carcinoma (EEC), esophageal cancer, gastric adenocarcinoma (GAC), and colon adenocarcinoma. The findings of this study suggest that targeting the METTL1–CDK4 axis could offer a therapeutic avenue for managing these cancer types, warranting further investigation into the molecular mechanisms underlying this interaction [14]. Zhang et al. focused on the intricate relationship between m6A modifiers and transcription factors (TFs) in the context of tumor development. This study revealed that TFs play pivotal roles in regulating gene expression through both transcriptional and posttranscriptional mechanisms. This dual regulatory capacity underscores the complexity of gene expression modulation within tumorigenic processes, suggesting that m6A modifications could influence the transcriptional landscape by altering the activity of specific transcription factors. These findings imply that understanding the interplay between m6A modifiers and TFs may provide valuable insights into the molecular underpinnings of cancer progression and potential therapeutic targets [15]. In their research, Xie et al. highlighted a significant correlation between elevated expression of the transcription factor homeobox-D1 (HOXD1) and adverse prognostic outcomes in various malignancies. This association was notably observed across multiple cancer types, including GAC, uterine corpus EEC, pheochromocytoma, and paraganglioma. These findings suggest that higher levels of HOXD1 may serve as potential biomarkers for poor prognosis, emphasizing the need for further investigation into its role in cancer pathophysiology and treatment response. The implications of these results are crucial for understanding the molecular mechanisms underlying cancer progression and for developing targeted therapeutic strategies [16].

These findings underscore the importance of m6A RNA methylation and its regulatory factors in the pathogenesis of GC and other malignancies.

## 4. GC Cell Line Experiments

The *MYC* proto-oncogene and bromodomain-containing protein 4 (BRD4) exert their effects by modifying the expression of key cancer-related genes. Studies have demonstrated its oncogenic activity in a wide array of cancers, including acute myeloid leukemia and gastric, lung, bladder, ovarian, colorectal, and pancreatic cancers. In a study by Kotekar et al., one notable mechanism involved the interaction of METTL3 with BRD4, a crucial regulator of gene transcription. The peptidyl-prolyl isomerase Pin1, a phosphorylation-directed proline isomerase, interacts with BRD4 phosphorylated at Thr204, positively regulating its stability. Pin1 subsequently isomerizes BRD4 at the nearby Pro205 residue, facilitating the interaction between BRD4 and CDK9 and ultimately promoting BRD4-mediated transcription in GC cells. Further research is necessary to fully elucidate the intricate interplay between METTL3, Pin1, and BRD4 in the context of various cancers. However, these findings highlight the critical role of METTL3 in cancer development [17].

In addition to BRD4 and c-MYC, heat shock protein 47 (HSP47) is another significant player in GC progression. In a study by Lee et al., HSP47 mRNA and protein expression levels were significantly elevated in GC tissues and cell lines compared with those in normal gastric mucosa. The silencing of HSP47 via siRNA results in reduced proliferation, migration, invasion, and wound repair capabilities in GC cells. Notably, in HSP47 siRNA-transfected GC cells, the expression of the metastasis-promoting gene matrix metallopeptidase-7 (*MMP-7*) was significantly downregulated. Interestingly, the regulation of *HSP47* appears to involve epigenetic mechanisms, as most noncancerous gastric tissues and the SNU-216 GC cell line, which exhibit low levels of HSP47, possess methylations in the *HSP47* promoter region. These findings underscore the complex interplay between epigenetic changes and gene expression in the context of GC [18].

The PR domain-containing (PRDM) family encompasses a variety of proteins, including PRDM14, which has garnered attention because of its significant role in GC. In a recent study by Li et al., both cell lines and patient tissues from individuals diagnosed with GC exhibited notable overexpression of PRDM14. Silencing the expression of *PRDM14* has been shown to induce apoptosis, halt the cell cycle, and inhibit the proliferation and migration of GC cells. These functional analyses indicate that PRDM14 plays a crucial role in epigenetic regulation by controlling several DNA methyltransferases and TFs. Moreover, a prognostic model based on differentially expressed genes, which incorporates PRDM14, has demonstrated robust predictive ability for patient outcomes. By utilizing the PRDM14 risk score alongside age and sex nomograms, researchers have been able to calculate survival probabilities for patients with greater accuracy. Further investigations into the therapeutic implications of PRDM14 have revealed a favorable correlation between its expression and sensitivity to various small-molecule medications. Notably, among these compounds are TPCA-1, an inhibitor of the IκB kinase subunit IKKβ (IKK2), which reduces the production of several proinflammatory cytokines and impedes NF-κB signaling; PF-56227, a well-established focal adhesion kinase (FAK) inhibitor; mirin, a compound characterized as 6-(4-hydroxyphenyl)-2-thioxo-2,3-dihydro-4(1H)-pyrimidinone; and linsitinib, an experimental drug candidate aimed at treating diverse cancer types. These findings underscore the potential of PRDM14 not only as a biomarker for prognosis but also as a target for therapeutic intervention in the treatment of GC [19].

Cell lines serve as pivotal in vitro model systems in a variety of medical research fields, notably in drug discovery and fundamental cancer research. Their primary advantage lies in their ability to provide an inexhaustible supply of the biological materials necessary for experimentation and investigation. When established under optimal conditions with appropriate controls, authenticated cancer cell lines typically retain the majority of the genetic characteristics of the original tumors from which they were derived. This assertion is substantiated by comparative analyses of the genomic data from various cancer cell lines against the findings derived from examining tumor tissue samples cataloged in The Cancer Genome Atlas (TCGA) database. Accessibility to extensive, publicly available cell line databases has significantly increased the precision of characterizing the cellular and molecular alterations within these lines. Moreover, the ability to establish specific culture conditions, such as three-dimensional (3D) cocultures that mimic the in vivo environment, underscores the growing importance of cancer cell lines in advancing medical research disciplines. However, it is imperative to acknowledge that the utility of cell lines is contingent upon their appropriate application. Ensuring data reproducibility and quality is essential to avoid misleading conclusions and adverse outcomes in research. Therefore, researchers must adhere to stringent protocols and guidelines when utilizing cell lines to preserve their integrity and reliability. In conclusion, while cancer cell lines represent a cornerstone of contemporary medical research, their effective application demands rigorous standards to fully harness their potential in contributing to our understanding of cancer biology and the development of therapeutic interventions [20].

## 5. In Vivo and In Vitro Experiments

In a study by Mohebbi et al., the interplay between lncRNAs and histone-modifying polycomb proteins played a critical role in the regulation of gene expression, particularly through the enhancement of the enzymatic functions of Zeste homolog 2 (EZH2). As a catalytic component of polycomb repressive complex 2 (PRC2), EZH2 is instrumental in the trimethylation of the 27th lysine residue on histone H3 (H3K27), a modification that is central to the epigenetic silencing of gene expression. This mechanism positions EZH2 as a significant oncogene in GC. Notably, lncRNAs that interact with EZH2 can facilitate the recruitment of this protein to the promoter regions of various tumor suppressor genes, thereby leading to their transcriptional inactivation. Evidence from in vitro and in vivo models of GC has demonstrated that the interactions between EZH2 and these lncRNAs modulate a range of biological processes, including drug resistance, cellular migration and invasion, metastatic progression, and the regulation of the cell cycle. Consequently, understanding the molecular dynamics of EZH2–lncRNA interactions is crucial for elucidating their contributions to the pathogenesis and progression of GC [21].

The exploration of epigenetic modulators offers promising insights into novel therapeutic strategies for managing GI cancers characterized by mutations in the tumor protein P53 (TP53). These mutations are known to facilitate tumorigenic processes, primarily by enabling the evasion of cellular senescence—a critical mechanism that serves as a natural barrier to cancer progression. A recent study by Wang et al. underscores the importance of targeting histone methylation as a pivotal approach in cancer treatment, particularly for tumors harboring TP53 mutations, which often exhibit enhanced resilience and persistence. A notable advancement in this domain is the utilization of QC6352, a selective inhibitor of lysine demethylase 4C (KDM4C), which has demonstrated efficacy in inducing cellular senescence in GC cells with *TP53* mutations. The application of QC6352, in conjunction with senescence-specific killing compound 1 (SSK1), represents a strategic “one-two punch” therapeutic regimen that effectively targets and eradicates tumor cells harboring TP53 mutations. This combination therapy not only disrupts the ability of the tumor to withstand therapeutic interventions but also promotes the reactivation of senescence pathways, thereby increasing the overall efficacy of cancer treatment. Continued research into the mechanistic roles of epigenetic modulators such as QC6352 is essential for developing innovative strategies aimed at overcoming the challenges posed by TP53 mutations in GI cancers [22].

The role of the significant antisense function 1B histone chaperone (ASF1B) in GC has garnered increasing attention because of its association with adverse clinical outcomes. In recent investigations by Zhao et al., both in vitro and in vivo, elevated levels of ASF1B not only correlated with poor prognostic indicators but also actively promoted the malignant characteristics of GC, suggesting potential therapeutic interventions. A pivotal transcription factor, Forkhead box protein M1 (FOXM1), has been identified as a key regulator of ASF1B expression and functions through direct interaction with the promoter region of the *ASF1B* gene. This regulatory mechanism elucidates the pathways through which FOXM1 influences GC progression. Notably, the administration of the FOXM1 inhibitor thiostrepton has shown dual efficacy: it not only impedes the development of GC but also leads to a significant reduction in ASF1B levels. Furthermore, ASF1B appears to regulate the transcription of the mitochondrial protein peroxiredoxin 3 (*PRDX3*) in a manner that is contingent upon FOXM1 activity, highlighting a crucial interplay between these proteins in the context of GC biology. These findings underscore the potential of targeting the ASF1B–FOXM1 axis as a therapeutic strategy in the management of GC [23].

A meticulous analysis of transcriptome datasets performed by Wu et al. revealed a significant reduction in the expression levels of homeodomain-interacting protein kinase 3 (HIPK3) in platinum-resistant tumors, which are closely linked to the resistance observed in patients receiving platinum-based therapies for GC. Both in vitro and in vivo experiments have indicated that the downregulation of *HIPK3* exerts effects contrary to those of *HIPK3* overexpression, notably leading to increased tumor proliferation and increased metastatic potential. Further investigations revealed that the transcriptional regulator myocyte enhancer factor 2C (MEF2C) is a key player in the downregulation of the morphogenesis regulator microtubule-associated protein 7 (MAP7), a process mediated through ubiquitination mechanisms. These findings underscore the critical role of HIPK3 in tumor biology and suggest potential therapeutic targets for overcoming resistance in GC treatment [24].

## 6. A Machine Learning Method

Acetyl-CoA acetyltransferase 2 (ACAT2), a member of the acetyl-CoA thiolase family, is expressed at significantly higher levels in GC tissues than in normal tissues. This upregulation of *ACAT2* is associated with critical implications for GC cell behavior, as evidenced by the profound inhibition of both motility and proliferation following the knockdown of *ACAT2*. Furthermore, in a study by Zhang et al., the histone methyltransferase SETD7, which plays a vital role in the maintenance and proliferation of GC cells, demonstrated markedly reduced transcriptional activity upon depletion of ACAT2. These findings suggest that the protumoral functions of ACAT2 are strongly dependent on SETD7. Additionally, SETD7 more efficiently ubiquitinates Yes-associated protein 1 (YAP1) in the absence of ACAT2. This reduced ubiquitination prevents the proteasomal degradation of YAP1, leading to the accumulation of the YAP1 protein. The resulting increase in *YAP1* expression significantly activates the YAP1/TAZ-TEAD1 signaling pathway, which consequently enhances the malignant phenotypes associated with GC cells. These findings collectively indicate that ACAT2 plays a pivotal role in promoting GC progression through its regulatory effects on SETD7 and subsequent modulation of the YAP1 signaling pathway [25].

The epithelial–mesenchymal transition (EMT) mechanism, which is influenced by miRNAs, has been identified as a significant contributor to the metastatic potential of tumor cells. The process of EMT facilitates the transition of epithelial cells into a mesenchymal phenotype, thereby enhancing their migratory and invasive capabilities. Consequently, the inhibition of EMT represents a promising therapeutic strategy aimed at mitigating the aggressive biological characteristics exhibited by tumor cells. A recent study by Sahib et al. suggested that targeting this pathway may lead to improved patient survival rates by reducing the likelihood of metastasis. Thus, a deeper understanding of the interplay between miRNAs and the EMT axis could be pivotal in developing effective interventions for cancer treatment [26].

Razavi-Mohseni et al. presented a novel machine-learning approach designed to identify the TFs that regulate various subtypes of GC through the analysis of ATAC-seq data. These findings revealed a significant association between the amplification of *MAPK9* and the deletion of *GATA4*, both of which contribute to the dysregulation of these critical TFs in GC. Furthermore, they successfully identified specific TFs that drive the mesenchymal state, including RUNX2, ZEB1, SNAI2, and the AP-1 dimer, alongside those associated with the epithelial state, namely, GATA4, GATA6, KLF5, HNF4A, FOXA2, and GRHL2. Comparative analyses with bulk and single-cell RNA-seq datasets indicate that mesenchymal-like GCs exhibit activated fibroblast-like epigenomic and expression profiles. Notably, differential accessibility of DNA cis-regulatory regions that flank upregulated mesenchymal genes appears to be linked to the activation of the mesenchymal fibrotic pathway. This research offers valuable insights into the molecular mechanisms underlying the heterogeneity of GC subtypes and highlights the potential applications of this approach in other cancers [27].

## 7. A Network-Based Integrative Method

Enhancer RNAs (eRNAs) are increasingly recognized as crucial regulators of gene expression. Recent investigations conducted by Bahrami et al. into the transcriptomic and epigenomic alterations associated with GC have employed a network-based integrative approach to elucidate these relationships. Notably, a central eRNA, ENSR00000272060, has been shown to be significantly correlated with various clinicopathological characteristics. This particular eRNA is markedly more highly expressed in tumor tissues than in nontumor counterparts, suggesting its potential role as a biomarker for GC. Furthermore, the analysis revealed that certain eRNAs presented genetic variations across the samples studied, indicating their association with the progression and staging of the disease. These findings underscore the importance of eRNAs not only in the regulatory networks of gene expression but also as potential indicators of disease pathology in GC [28].

Methyl-CpG binding domain protein 3 (MBD3) has emerged as a pivotal epigenetic regulator that plays a significant role in the pathology of various malignancies, particularly in GC. Wang et al. reported that *MBD3* is aberrantly expressed in GC tissues and cells compared with their normal counterparts, revealing an elevated expression profile that is associated with poor prognostic outcomes in patients diagnosed with GC. Specifically, the upregulation of *MBD3* enhances the migratory, invasive, and proliferative capacities of GC cells, thereby facilitating tumor metastasis and progression. The mechanism underlying this phenomenon involves the activation of the phosphoinositide 3-kinase (PI3K)/AKT signaling pathway, which is known to promote EMT, a critical process in cancer metastasis. Furthermore, MBD3 has been shown to upregulate the expression of actin γ1 (*ACTG1*), which consequently contributes to increased motility and proliferation of GC cells. These findings underscore MBD3 as a potentially significant therapeutic target in the management of GC, warranting further investigation into its role in tumor biology and its implications for patient prognosis [29].

## 8. Analysis of the Immunological Landscape

In a comprehensive investigation by Ouyang et al. of the unfolded protein response (UPR), close functional linkages and significant enrichment were identified within the protein modification, transport, and RNA degradation pathways associated with 113 UPR-related genes. By employing unsupervised clustering methodologies, two distinct molecular subtypes were delineated, revealing substantial discrepancies in gene expression profiles as well as prognostic outcomes. Further exploration of the immunological landscape suggested that the UPR may play a pivotal role in shaping the immune microenvironment within tumors. To enhance its predictive ability, an independent nomogram was developed, and a prognostic signature comprising seven UPR-related genes was established and validated. Notably, the gene encoding insulin-like growth factor binding protein 1 (IGFBP1) emerged as the most significant contributor within this prognostic framework, as it presented the highest weight coefficient. This gene was found to promote the malignant phenotype of GC cells, indicating its potential as a promising therapeutic target in the management of this disease [30].

Cytokines, a category of proteins integral to the processes of inflammation and the immune response, have garnered significant attention in recent research by Reyes et al. for their potential implications in the pathogenesis of various cancers, including GC. The involvement of cytokines in cancer progression is complex, as they play both proinflammatory and anti-inflammatory roles, influencing the tumor microenvironment (TME) and cellular behavior. Epigenetic modifications, including DNA methylation, histone protein alterations, and the regulation of noncoding RNAs, play pivotal roles in the expression of these inflammatory mediators. These modifications can lead to the aberrant overexpression or silencing of critical genes associated with GC. This dysregulation can have far-reaching consequences, impacting not only the genes directly involved in GC but also those that participate in key cytokine-related signaling pathways. The interplay between cytokines and these epigenetic mechanisms underscores the importance of understanding the molecular underpinnings of inflammation in the context of cancer biology, particularly in relation to the etiology and progression of GC [31].

One significant epigenetic component associated with various physiological processes, including osteoclast differentiation and adipogenesis, is carboxypeptidase X member 1 (CPXM1). Recent gene enrichment analyses conducted by Gu et al. suggested that *CPXM1* plays a pivotal role in regulating the development and progression of GC through key signaling pathways, specifically the transforming growth factor-beta (TGF-β) and phosphoinositide 3-kinase-Akt (PI3K-AKT) pathways. The expression of *CPXM1* is linked to increased proportions of stromal and immune cells within the TME. Notably, the number of M2 macrophages was directly correlated with *CPXM1* expression, whereas the percentage of plasma cells was inversely related to CPXM1 levels. These findings underscore the complex role of CPXM1 in modulating immune responses and its potential implications in cancer biology [32].

## 9. Potential Biomarkers for Different Cancers

In various malignancies, including GC, the expression of *EGLN3*, which belongs to the EGL-9 family of hypoxia-inducible factors, has been reported to be significantly downregulated in a study by Cai et al. This reduction in *EGLN3* expression in GC tissues correlates with poorer patient prognosis, suggesting its potential role as a prognostic biomarker. Research has indicated that hypermethylation of *EGLN3* disrupts transcriptional balance, exacerbating the progression of GC by promoting increased tumor invasion and lymph node metastasis. Conversely, the restoration of *EGLN3* expression notably inhibited both the proliferation and metastatic potential of GC cells. Moreover, EGLN3 exerts its anti-malignant effects independent of its hydroxylase activity by attenuating the activation of the NF-κB signaling pathway, a process mediated by Jumonji C domain-containing protein 8. These findings underscore the critical role of EGLN3 in GC pathophysiology and highlight its potential as a therapeutic target for intervention in GC progression [33].

The hypermutator phenotype identified as microsatellite instability (MSI) arises from a dysfunctional DNA mismatch repair system, leading to an accumulation of mutations that contribute to tumorigenesis. A study by Cao et al. revealed a strong association between MSI and several malignancies, including CRC, GC, and endometrial cancer, positioning MSI as a critical factor in understanding tumor biology and patient outcomes. Recent advancements in computational biology have facilitated the exploration of MSIs as biomarkers for immune checkpoint therapies, providing a pathway for personalized cancer treatment. The MSI-XGNN framework utilizes a combination of DNA methylation profiles and bulk RNA sequencing data to predict MSI status in cancerous tissues. Through this innovative approach, six key MSI indicators were identified, comprising two genes—*MSH4* and *RPL22L1*—and four specific methylation probes: EPM2AIP1|MLH1:cg27331401, LNP1:cg05428436, and TSC22D2:cg15048832. This ensemble forms a robust feature subset that enhances the accuracy of MSI predictions. The identified MSI indicators were significantly correlated with immunotherapy-responsive features of the TME. Notably, these markers align with established parameters such as tumor mutation burden and neoantigen presence. Moreover, they are strongly associated with immunological checkpoint molecules, including cytotoxic T-lymphocyte antigen-4 (CTLA-4) and programmed cell death-1 (PD-1), which are instrumental in the effectiveness of immunotherapy. The implications of the MSI-XGNN framework extend beyond predictive capabilities; they also contribute to a deeper understanding of the immunological landscape of the TME. The correlation of MSI indicators with immune checkpoints signifies their potential utility in stratifying patients for immunotherapeutic interventions, thus paving the way for more tailored treatment approaches in oncology. The emergence of the MSI-XGNN framework represents a significant advancement in the field of cancer genomics, offering a reliable method for predicting MSI status through integrated data analysis. As research continues to unveil the complexities of tumor biology, incorporating MSI as a biomarker for immunotherapy will increase the efficacy of treatment strategies and improve patient outcomes in various malignancies [34].

Globally, GC and CRC rank among the leading contributors to cancer-related mortality. Despite advancements in therapeutic interventions and a deeper understanding of the molecular mechanisms underlying these malignancies, the overall survival rates for affected patients remain suboptimal. Recent research conducted by Zabeti et al. highlighted the significant role of lncRNAs in the initiation, progression, and metastasis of various cancers, including GC and CRC. LncRNAs influence gene expression through a multitude of mechanisms, including interactions with proteins and miRNAs, as well as through epigenetic modifications. By functioning as precursors to miRNAs or pseudogenes, lncRNAs can regulate gene expression at both the transcriptional and posttranscriptional levels. This intricate regulatory network underscores the potential of lncRNAs as key players in cancer biology and emphasizes the need for further investigation into their therapeutic implications in improving patient outcomes in patients with GC and CRC [35].

Sirtuin 7 (SIRT7), a notable member of the sirtuin family, plays a critical role in a myriad of cellular functions. Predominantly localized within the nucleolus, SIRT7 exhibits a diverse range of enzymatic activities that are essential for cellular homeostasis, according to Lagunas-Rangel et al. SIRT7 is integral to several key processes, including the expression of rRNA, DNA damage repair mechanisms, the cellular stress response, and the compaction of chromatin. Recent research has elucidated the connection between SIRT7 and various pathologies, particularly obesity, cardiovascular diseases, bone disorders, and, notably, cancer. Evidence indicates that *SIRT7* is often overexpressed in several malignancies, including HCC, GC, lung adenocarcinoma (LUAD), prostate adenocarcinoma, clear cell renal cell carcinoma, and breast cancer. The overexpression of *SIRT7* in these cancers suggests its pivotal role in the regulation of cancer-related genes through epigenetic modifications. Furthermore, cancer cells exploit SIRT7 to adapt to stressful microenvironments, thereby increasing their growth and metabolic processes via ribosome biogenesis. These findings underscore the importance of SIRT7 in cancer biology and its potential as a therapeutic target for intervention in various malignancies [36].

Metastatic-associated lung adenocarcinoma transcript 1 (MALAT1) is a widely studied long noncoding RNA (lncRNA) known for its significant role in oncogenesis and cancer progression. In a study by Zhang et al., elevated expression levels of MALAT1 were strongly associated with various cancer-related phenomena, such as carcinogenesis, tumor progression, metastasis, drug resistance, and overall treatment outcomes in solid tumors. This lncRNA has been implicated in the pathogenesis of numerous solid malignancies, including GC, CRC, osteosarcoma, gliomas, and pancreatic and breast cancers, where its overexpression is frequently observed. MALAT1 employs several regulatory mechanisms, including the sponging of miRNAs and epigenetic modifications, to influence a multitude of target genes, paralleling its functions in solid tumors. Notably, the specific miRNAs with which MALAT1 interacts differ from those identified in solid tumors, indicating a complex and varied regulatory landscape that warrants further investigation in the context of different cancer types. This highlights the need for further exploration into the molecular mechanisms underlying the role of MALAT1 in oncogenesis and potential therapeutic interventions targeting its pathways [37].

Table 1 summarizes the pivotal home messages regarding epigenetic regulatory processes derived from studies conducted from 2023 to 2024.

## 10. Other GI Tumors

Intestinal metaplasia (IM) and intraepithelial neoplasia (IEN) are recognized as significant precursors in the progression of GC. A recent study by Liao et al. highlighted a marked difference in the prevalence of precancerous gastric cardia lesions (PGCLs) in various geographical regions, with individuals residing in high-incidence areas exhibiting a more pronounced risk (26.7%) than their counterparts in non-high-incidence areas (13.5%). The investigation of differentially methylated probes (DMPs) and differentially expressed genes (DEGs) has shed light on the gradual alterations in DNA methylation and gene expression that accompany the progression from type I IM to type II IM and ultimately to type III IM, with findings indicating a significant increase in both DMPs and DEGs at each stage: type I IM (DMP = 210, DEG = 24), type II IM (DMP = 3402, DEG = 129), and type III IM (DMP = 3735, DEG = 328). Notably, IEN exhibited the greatest degree of change, with DMPs and DEGs peaking at (DMP = 47,373 and DEG = 2278). Furthermore, the study revealed aberrant promoter methylation in three DEGs that were exclusively shared between type III IMs and IENs. Among these DEGs, *OLFM4* expression was found to be present in IMs and significantly increased in IENs (*p* < 0.001), underscoring its potential role in the early stages of gastric carcinogenesis. These data emphasize the critical need for further research into the molecular mechanisms underlying IM and IEN, as they represent pivotal points in the continuum leading toward GC [38].

The SMYD family comprises a group of proteins characterized by their lysine methyltransferase activity, with SMYD4 being a notable member. Investigations by Olivera Santana et al. into various tumors have revealed a generally low incidence of mutations within SMYD genes; however, the specific mutations identified in *SMYD4* do not exhibit sufficient discriminatory power to serve as reliable biomarkers for oncological diagnosis or prognosis. In terms of copy number alterations (CNAs), all the assessed tumors presented consistent homozygous deletions and downregulation of *SMYD4* expression. Notably, compared with normal tissue, tumor samples presented markedly lower expression levels of *SMYD4*, with the exception of GAC, whose expression levels were elevated. Additionally, a positive correlation was established between SMYD4 expression, CNAs, and mRNA levels, indicating a potential regulatory relationship. Conversely, *SMYD4* expression was negatively correlated with that of other SMYD family members, suggesting complex interactions among these proteins. Importantly, the clinical outcomes revealed that patients with low *SMYD4* expression in GAC and LUAD patients experienced significantly poorer overall survival rates than those with higher *SMYD4* expression levels. Collectively, these findings substantiate the role of SMYD4 as a potential tumor suppressor in various malignancies, reinforcing its importance in cancer biology and patient prognosis [39].

Aberrations in posttranslational modifications of ribosomal proteins play a significant role in cancer biology. Among these, the lysine methyltransferase SMYD5 has emerged as a key player in the trimethylation of rpL40 at lysine 22 (rpL40K22me3). Elevated levels of SMYD5 and rpL40K22me3 have been observed in GAC patient samples, suggesting a correlation between these markers and tumor aggressiveness. Notably, the increased expression of these proteins does not correlate well with clinical outcomes, highlighting the need for further investigation into their biological significance. Using both familial and sporadic mouse models of malignant GAC, the effects of SMYD5 ablation were assessed in vivo by Park et al. The progression of peritoneal carcinomatosis was monitored following the genetic disruption of *SMYD5*, while the impact on GAC xenografts derived from patient samples and human cancer cell lines was evaluated in vitro. These findings indicate that ablation of SMYD5 significantly inhibits disease progression in mouse models, effectively preventing the dissemination of MCs, including those associated with the development of peritoneal carcinomatosis. Moreover, the suppression of *SMYD5*-mediated methylation of rpL40 enhances the sensitivity of GAC cells to phosphoinositide 3-kinase (PI3K) and mechanistic targets of rapamycin (mTOR) inhibitors. These data underscore the pivotal role of SMYD5 in the malignant evolution of GAC through its regulation of rpL40 trimethylation. The elevation of SMYD5 and rpL40K22me3 in clinical samples is correlated with a more aggressive disease phenotype, making them potential biomarkers for GAC prognosis. Furthermore, the therapeutic implications of targeting *SMYD5* may provide novel avenues for enhancing the efficacy of existing cancer treatments. In conclusion, the regulation of rpL40 trimethylation by SMYD5 is integral to the progression of GAC. The elucidation of this pathway presents promising opportunities for therapeutic intervention, warranting further research to explore the clinical application of SMYD5 inhibitors in GAC management [40].

The term “esophagogastric junction cancer” (EJC) refers to the malignant growth that occurs at the anatomical convergence of the esophagus and stomach. Recently, Liu et al. identified a novel tumor suppressor gene known as *TUSC1*, which has been implicated in the pathogenesis of various cancer types. Within the context of EJC, the expression of *TUSC1* is significantly downregulated, suggesting a potential role in tumorigenesis. Experimental evidence indicates that the overexpression of *TUSC1*, in conjunction with the application of 5-AZA-2 therapy, effectively inhibits the malignancy of EJC cells. Furthermore, low levels of methylation in EJC tissues correlate with increased *TUSC1* expression. Notably, the upregulation of *TUSC1* has been shown to repress *MDM2* expression while simultaneously activating the p53 signaling pathway. However, the antiproliferative, antimigratory, and anti-invasive effects of *TUSC1* overexpression are diminished when this critical pathway is inactivated. These findings underscore the importance of TUSC1 in EJC and highlight its potential as a therapeutic target in cancer treatment [41].

The term “GI malignancies” encompasses a diverse range of cancers that pose significant challenges to global health systems. These malignancies, which include cancers of the esophagus, stomach, liver, pancreas, and colorectal regions, are associated with high morbidity and mortality rates worldwide. A critical component of the body’s defense against these cancers is immune surveillance, a process that is largely mediated by the major histocompatibility complex (MHC). The MHC plays a pivotal role in the immune system’s ability to recognize and eliminate tumor cells, facilitating the presentation of tumor antigens to T cells. However, according to Tovar Perez et al., the regulation of MHC gene expression is complex and subject to dynamic epigenetic modifications. These modifications can significantly influence the pathogenicity and functional outcomes of immune responses against tumors. Consequently, understanding the interplay between epigenetic alterations and MHC expression is essential for developing effective therapeutic strategies to enhance immune responses against GI malignancies [42].

Table 2 provides a comprehensive overview of the essential home messages regarding epigenetic regulatory processes derived from studies conducted on various GI tumors from 2023 to 2024.

## 11. Conclusions

lncRNAs such as LINC00853 and LINC01266 have emerged as significant factors in the dysregulation of gene expression and epigenetic alterations observed in GC tumors. These lncRNAs contribute to the complex molecular landscape that characterizes cancer pathology, thereby influencing tumor behavior and patient outcomes. Additionally, circRHOT1 has been a focal point of research because of its regulatory roles in oncogenesis and tumor progression. The evidence suggests that the overexpression of *KAT5* and *GPX4* can mitigate the antiproliferative effects typically associated with the depletion of circRHOT1, highlighting a potential therapeutic target in GC treatment. Furthermore, mutations in m6A have been shown to play critical roles in various posttranscriptional processes, including splicing, RNA export, degradation, and translation of mRNAs. These alterations can significantly impact cancer pathophysiology, thereby contributing to the complexity of tumor biology and the development of targeted therapies. Understanding these molecular mechanisms is crucial for advancing treatment strategies for GC and improving patient prognosis.

The protein METTL3 has emerged as a significant factor in the oncogenic processes associated with GC. Its role is characterized primarily by the modulation of the expression of key cancer-related genes, particularly those encoding *Myc* and *BRD4*. METTL3 has been shown to interact directly with BRD4, a vital transcriptional regulator that plays an essential role in gene expression in cancer cells. This interaction facilitates BRD4-mediated transcriptional activity, thereby contributing to the proliferation and survival of GC cells. Furthermore, HSP47, a heat shock protein, has been implicated in the progression of GC. The regulation of HSP47 appears to be influenced by epigenetic mechanisms, as evidenced by the observation that most noncancerous gastric tissues and the SNU-216 GC cell line present markedly low levels of HSP47 expression. These findings suggest that understanding the intricate regulatory networks involving METTL3, BRD4, and HSP47 may provide valuable insights into the molecular underpinnings of GC and potentially reveal novel therapeutic targets.

EZH2 plays a pivotal role in epigenetic regulation by catalyzing the trimethylation of the 27th lysine residue on histone H3, a modification that is integral to the silencing of gene expression. The intricate molecular dynamics underlying the interactions between EZH2 and long lncRNAs are essential for elucidating their respective roles in the pathogenesis and progression of GC. Recent studies have highlighted the potential therapeutic benefits of QC6352, a selective inhibitor of lysine demethylase 4C, which has shown promise in inducing cellular senescence, specifically in GC cells harboring TP53 mutations. These findings suggest that targeting the epigenetic machinery involved in GC may provide a novel approach for therapeutic intervention, warranting further investigation into the mechanistic pathways influenced by EZH2 and its regulatory networks. Understanding these interactions could pave the way for the development of more effective strategies to combat GC and improve patient outcomes.

Enhancer RNAs serve as vital regulators of gene expression and play essential roles in the modulation of various biological processes. Recent studies have highlighted the importance of a specific enhancer RNA, ENSR00000272060, which has been found to be significantly correlated with clinicopathological characteristics in patients with GC. This correlation suggests that ENSR00000272060 may be integral to the progression and severity of this malignancy. Furthermore, MBD3, an epigenetic regulator, has emerged as a critical player in the pathology of malignancies, particularly in the context of GC. The interaction between MBD3 and enhancer RNAs such as ENSR00000272060 could provide valuable insights into the epigenetic mechanisms underlying tumorigenesis and may offer potential avenues for targeted therapeutic interventions in GC. Understanding the intricate relationships among these molecular players is crucial for advancing our knowledge of GC biology and improving patient outcomes.

lncRNAs have emerged as critical regulators of the initiation, progression, and metastasis of various malignancies, particularly GC. These lncRNAs contribute to the complex molecular landscape that defines tumor behavior and response to treatment. A comprehensive understanding of the interplay between epigenetic alterations and MHC expression is pivotal for the advancement of therapeutic strategies aimed at augmenting immune responses against GI cancers. By elucidating the mechanisms through which lncRNAs influence MHC expression and modulate immune evasion, researchers can develop targeted interventions that may improve patient outcomes in these malignancies. Thus, further investigation into the role of lncRNAs in the context of epigenetics and immune modulation is essential for informing innovative approaches to cancer therapy.

In recent years, the field of epigenetics has emerged as a crucial area of study, demonstrating that gene expression can be modified by environmental factors and lifestyle choices without altering the underlying DNA sequence. These findings have significant implications for understanding the etiology of various diseases, including cancer, cardiovascular disorders, and neurodegenerative conditions. As such, integrating epigenetic research into clinical practice is essential for several reasons. First, epigenetic mechanisms offer valuable insights into disease prevention and management. By understanding how environmental factors—such as diet, stress, and exposure to pollutants—can influence gene expression, healthcare professionals can develop personalized prevention strategies tailored to individual patients. This shift from a one-size-fits-all approach to a more nuanced understanding of patient-specific risk factors can lead to more effective interventions and improved health outcomes. Second, epigenetic research has the potential to revolutionize diagnostic practices. Traditional diagnostic methods often focus on genetic mutations, which may not fully capture an individual’s health status or disease risk. By incorporating epigenetic markers into diagnostic protocols, clinicians can gain a more comprehensive understanding of a patient’s biological state, enabling earlier detection and more accurate prognoses. Furthermore, the therapeutic application of epigenetics is rapidly evolving. Epigenetic therapies, such as those targeting DNA methylation or histone modification, are being explored in clinical trials for various diseases. By integrating this cutting-edge research into clinical practice, healthcare providers can offer new treatment modalities that are aimed at reversing disease processes rather than merely alleviating symptoms. In conclusion, the integration of epigenetic research into clinical practice is not merely beneficial; it is imperative. As our understanding of the epigenetic landscape continues to grow, the potential for improved disease prevention, more accurate diagnostics, and innovative therapies becomes increasingly attainable. It is crucial for the medical community to embrace this paradigm shift, ensuring that patients receive the most advanced and effective care possible. By doing so, we can move towards a future where health care is truly personalized and optimally aligned with the complexities of human biology.

## Figures and Tables

**Table 1:** Epigenetic regulatory processes in GC.

Author and the Year That Paper Was Published	GC’s Epigenetic Regulatory Mechanisms
Xu P et al., 2024 [3]	An important part of the growth and development of GC is played by *LINC00853*.
Hua H et al., 2023 [4]	The significance of functional lncRNAs in cancer as novel therapeutic targets since they can precisely regulate protein–protein interactions.
Xie M et al., 2023 [5]	It is possible that *ASH1L-AS1* will be a new target for GC therapies and clinical diagnostics.
Askari N et al., 2023 [6]	To provide useful prospective biomarkers for the prognosis of patients with GC, a network of drug–gene interactions for newly identified genes was projected.
Wang H, et al., 2023 [7]	A potential target for GC therapy is cirRHOT1.
Wang M, et al., 2023 [11]	The manner in which m6A causes GI cancer was elucidated.
Wu X et al., 2024 [12]	FTO stimulated the growth of GC by means of the circFAM192A/*SLC7A5* axis in a manner that was dependent on m6A.
Liu T et al., 2023 [13]	*METTL3* controls lncRNA SNHG7’s m6A methylation level and expression in GC caused by MNNG exposure, indicating that *SNHG7* may be a useful biomarker or therapeutic target for GC.
Zhang C et al., 2023 [14]	Potential therapeutic target for malignancies of the digestive system: the *METTL1/CDK4* axis
Xie R et al., 2024 [16]	The *IGF2BP3*/*OIP5-AS1*/*hnRNPA1* axis may offer a possible diagnostic or prognostic target for GC. *OIP5-AS1* is an oncogenic m6A-modified lncRNA in GC.
Kotekar et al., 2023 [17]	It was discovered that *BRD4* controlled the growth of GC cells.
Lee J et al., 2024 [18]	Targeting *HSP47*—possibly by methylating its promoter—could be a helpful new treatment approach for GC.
Li X et al., 2024 [19]	The progression of GC is positively regulated by *PRDM14*.
Mohebbi H et al., 2024 [21]	For the development of innovative targeted therapeutics for GC, *EZH2*-interacting lncRNAs are intriguing targets.
Wang K et al., 2023 [22]	A potential ‘one-two punch’ therapy approach for the more aggressive forms of GC is represented by QC6352 and the senolytic agent SSK1.
Zhao Z et al., 2024 [23]	Therapeutic targeting of the *FOXM1-ASF1B-PRDX3* axis may be possible for the treatment of GC.
Wu QN et al., 2024 [24]	*HIPK3* level monitoring may be used as a platinum resistance and GC malignancy biomarker.
Zhang M et al., 2024	The molecular basis and pro-tumoral actions of *ACAT2* in GC cells were demonstrated, indicating that *ACAT2* may be a viable target for GC therapy.
Sahib AS et al., 2023 [26]	Lack of particular attention to the regulation of EMT in particular types of tumor cells is one of the gaps in our understanding of cancer metastasis.
Razavi-Mohseni M et al., 2024 [27]	TF activity in GC was mapped, and it was shown how copy number-driven changes shape epigenomic regulatory programs and may be important contributors to the heterogeneity and advancement of GC.
Bahrami B et al., 2024 [28]	The findings indicate that the quantity of eRNA analyzed varies throughout the samples. It implies that we might be able to use it to identify the illness at an earlier stage.
Wang H et al., 2024 [29]	Through a nonenzymatic mechanism, *EGLN3* has the ability to impede the spread of GC cells, providing insight into the intricate nature of GC growth.
Ouyang W et al., 2024 [30]	By upregulating *ACTG1* via PI3K/AKT signaling activation, *MBD3* increased migration, invasion, proliferation, and EMT in GC cells and may be a useful target for prognostic and diagnostic purposes.
Reyes ME et al., 2024 [31]	The development of a UPR-related gene classifier and risk signature for GC survival prediction revealed *IGFBP1* to be a critical driver of the disease’s malignancy and a promising target for therapy.
Gu Q et al., 2023 [32]	Epigenetic modifications, particularly DNA hypermethylation, impact critical genes and immune response pathways, impacting angiogenesis, which is necessary for tumor development and metastasis in the progression of GC.
Cai F et al., 2024 [33]	As *CPXM1* controls the course of GC, it might be a new target for GC detection and therapy.
Cao Y et al., 2023 [34]	An effective and comprehensible deep learning model was given to predict MSI status and identify MSI markers that might be used for clinical MSI evaluation.
Zabeti Touchaei A et al., 2024 [35]	More research is required to determine the role that lncRNAs play in the management of GC and CRC.
Lagunas-Rangel FA. 2023 [36]	There was a discussion of the viability and difficulties involved in creating medications that can alter SIRT7 activity.
Zhang C. et al., 2024 [37]	Large-scale clinical trials should be conducted to further confirm the clinical value of *MALAT1*.

**Table 2:** Epigenetic regulatory processes in other GI tumors.

Author and the Year That Paper Was Published	Epigenetic Regulatory Mechanisms
Liao X. et al., 2024 [38]	Type III IM and IEN suggest biomarkers that may be useful in risk prediction and share comparable epigenetic and transcriptional characteristics in gastric cardiac tissue.
Olivera Santana BL et al., 2024 [39]	*SMYD4*’s persistent downregulation and correlation with the advancement of cancer highlight the biomarker’s potential utility.
Park J, et al., 2024 [40]	An epigenetic mechanism based on ribosomes promotes the development of malignant GAC and suggests that targeting *SMYD5* could be a component of combination therapy for the treatment of GAC.
Liu Z et al., 2024 [41]	*TUSC1* was frequently downregulated and methylation-regulated. By modulating the p53 pathway, it inhibited the cancerous development of EJC tumors, indicating its potential as an EJC diagnostic and treatment target.
Tovar Perez JE et al., 2024 [42]	Future research and treatment development could be greatly aided by the combination of epigenetic-targeted medicines and immunotherapies, which could improve clinical outcomes for patients with GI cancers.

## Data Availability

Not applicable.
